# Cancer associated thrombosis and mortality in patients with cancer stratified by khorana score risk levels

**DOI:** 10.1002/cam4.3437

**Published:** 2020-09-20

**Authors:** Alok A. Khorana, Nicole M. Kuderer, Keith McCrae, Dejan Milentijevic, Guillaume Germain, François Laliberté, Sean D. MacKnight, Patrick Lefebvre, Gary H. Lyman, Michael B. Streiff

**Affiliations:** ^1^ Cleveland Clinic Cleveland OH USA; ^2^ Advanced Cancer Research Group Seattle WA USA; ^3^ Janssen Scientific Affairs, LLC Titusville NJ USA; ^4^ Groupe d’analyse Ltée Montreal QC Canada; ^5^ Fred Hutchinson Cancer Research Center and University of Washington Seattle WA USA; ^6^ Division of Hematology Department of Medicine John Hopkins University School of Medicine Baltimore MD USA

**Keywords:** Cancer, clinical cancer research, medical oncology, risk assessment, risk model, Cancer, Venous thromboembolism

## Abstract

**Background:**

The Khorana score (KS) clinical algorithm is used to predict VTE risk in cancer patients. The study objective was to evaluate VTE and survival rates among patients newly diagnosed with cancer and stratified by KS in a real‐world population.

**Methods:**

Data from the Optum^®^ Clinformatics^®^ DataMart database between 01/01/2012–09/30/2017 was used to identify adults with ≥ 1 hospitalization or ≥ 2 outpatient claims with a cancer diagnosis (index date). Only patients who were initiated on chemotherapy or radiation therapy were included. Patients were classified based on KS (KS = 0, 1, 2 or ≥ 3). Time‐to‐first VTE and survival were evaluated from the index date to the earliest among end of data availability or insurance coverage, death, or 12 months post‐index using Kaplan‐Meier (KM) analyses.

**Results:**

A total of 2,488 (KS = 0); 2,125 (KS = 1), 1,074 (KS = 2), and 507 (KS ≥ 3) cancer patients were included. The 12‐month KM rates of VTE were 3.1%, 5.4%, 7.9%, and 14.9% (associated median time to VTE of 2.7, 3.0, 1.4, and 1.7 months) among KS = 0, 1, 2, and ≥ 3 cohorts, respectively. Corresponding adjusted hazard ratios (95% CIs) relative to the KS = 0 cohort were 1.72 (1.25‐2.38), 2.46 (1.73‐3.50), and 4.99 (3.40‐7.31) for the KS = 1, 2, and ≥ 3 cohorts, respectively (all *P* < .001). Regardless of KS, patients with VTE had significantly lower survival rates than those without.

**Conclusions:**

This real‐world claims‐based cohort study of newly diagnosed cancer patients showed significantly higher rates of VTE with increased KS, confirming its predictive ability. Moreover, VTE was associated with lower survival rates within each KS cohort.

## Graphical abstract

This cohort study evaluated the risk of VTE and survival rates among patients newly diagnosed with cancer and stratified by Khorana score (KS) in a real‐world population. The present findings indicate that the rates of VTE significantly increased with KS, confirming its predictive ability. Moreover, VTE was associated with lower survival rates within each KS cohort.

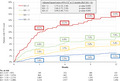

## 1 INTRODUCTION

Venous thromboembolism (VTE) is a common complication of cancer that includes deep vein thrombosis and pulmonary embolism.^1^, ^2^, ^3^ Cancer increases the risk of VTE by approximately four to seven fold,^1^, ^3^ and systemic therapy further increases this risk.^2^, ^3^ Moreover, cancer‐associated VTE is associated with worsened survival; studies have found that VTE was associated with a ≥ 2 fold increased risk of mortality in patients with cancer.^4^, ^5^, ^6^, ^7^

The risk of VTE varies widely among cancer patients.^8^ The Khorana score (KS) is an externally validated risk prediction clinical algorithm^9^, ^10^, ^11^, ^12^, ^13^, ^14^, ^15^, ^16^, ^17^, ^18^, ^19^, ^20^, ^21^, ^22^, ^23^ to assess the risk of VTE in patients with cancer. This score uses multiple independent predictors, including the tumor's primary site, pre‐chemotherapy platelet count, hemoglobin levels < 100 g/L or use of red blood cell growth factors, pre‐chemotherapy leucocyte count > 11 x 10^9^/L, and body mass index (BMI) ≥ 35 kg/m^2^.^24^ Although other scores have been developed,^10^, ^23^, ^25^, ^26^ these have either not been validated or not assessed for thromboprophylaxis efficacy. KS remains the most validated risk assessment tool for VTE and is endorsed by multiple clinical practice guidelines.^27^, ^28^, ^29^

Several clinical studies and post‐hoc analyses have shown that patients at high risk of VTE — identified using KS thresholds of ≥ 2 or ≥ 3 – can benefit from primary VTE prophylaxis.^23^, ^30^, ^31^, ^32^, ^33^ In these studies for risk‐stratified patients, the magnitude of the difference in VTE risk between the treatment and placebo arms was invariably larger than those reported in previous trials of unselected populations (ie without risk‐based selection).^34^, ^35^ Most recently, results from two randomized trials–CASSINI (rivaroxaban vs placebo)^36^ and AVERT (apixaban vs placebo)^37^—demonstrated the benefits of primary VTE prophylaxis with the direct oral anticoagulants rivaroxaban or apixaban in patients with cancer and a KS ≥ 2. The data generated by these studies recently led the American Society of Clinical Oncology (ASCO), the International Initiative on Thrombosis and Cancer (ITAC), and the International Society on Thrombosis and Haemostasis (ISTH) to offer rivaroxaban, apixaban, or low molecular weight heparin (LMWH) for primary VTE prophylaxis in ambulatory patients with cancer with KS of 2 or higher.^28^, ^29^, ^38^

The rates of VTE in patients diagnosed with cancer stratified by VTE risk, as assessed using a clinical prediction algorithm such as the KS, have never been studied in a large cohort representative of the US population. There is limited information on symptomatic VTE rates and how it may affect survival in patients with cancer particularly when stratified by KS. Therefore, the current study sought to assess VTE incidence and survival rates in a broad US population of patients newly diagnosed with cancer who received various cancer treatments across various strata of KS.

## 2 METHODS

### 2.1 Data source

Healthcare insurance claims from the Optum^®^ Clinformatics^®^ Data Mart database were used, with data from January 2012 through September 2017. Optum Clinformatics covers 12‒14 million annual lives of a large national managed care company affiliated with Optum in all census regions of the United States. It contains more than 36 months of historical data on patients (claims from commercial and Medicare Advantage plans including patients’ demographics, dates of eligibility, inpatient and outpatient visit claims, laboratory tests and results, and date of death). The database only contains de‐identified data that fully comply with the confidentiality requirements of the Health Insurance Portability and Accountability Act.

### 2.2 Study design

This study used a retrospective cohort study design with the index date defined as the date of the first claim for a cancer diagnosis with ≥ 6 months of continuous eligibility before the index date (ie baseline period). The 45‐day period following the index date was used to assess cancer treatment initiation to avoid patients with inappropriately delayed therapy starts. The risk stratification period was defined as the 28 days prior to the initiation of cancer treatment (this period may overlap with the baseline period) during which laboratory values were collected for the KS calculation. The follow‐up was defined as the period between the index date and the earliest of end of data availability, end of insurance coverage, death, or 12 months post‐index, whichever came first.

### 2.3 Study population

**Figure 1:**
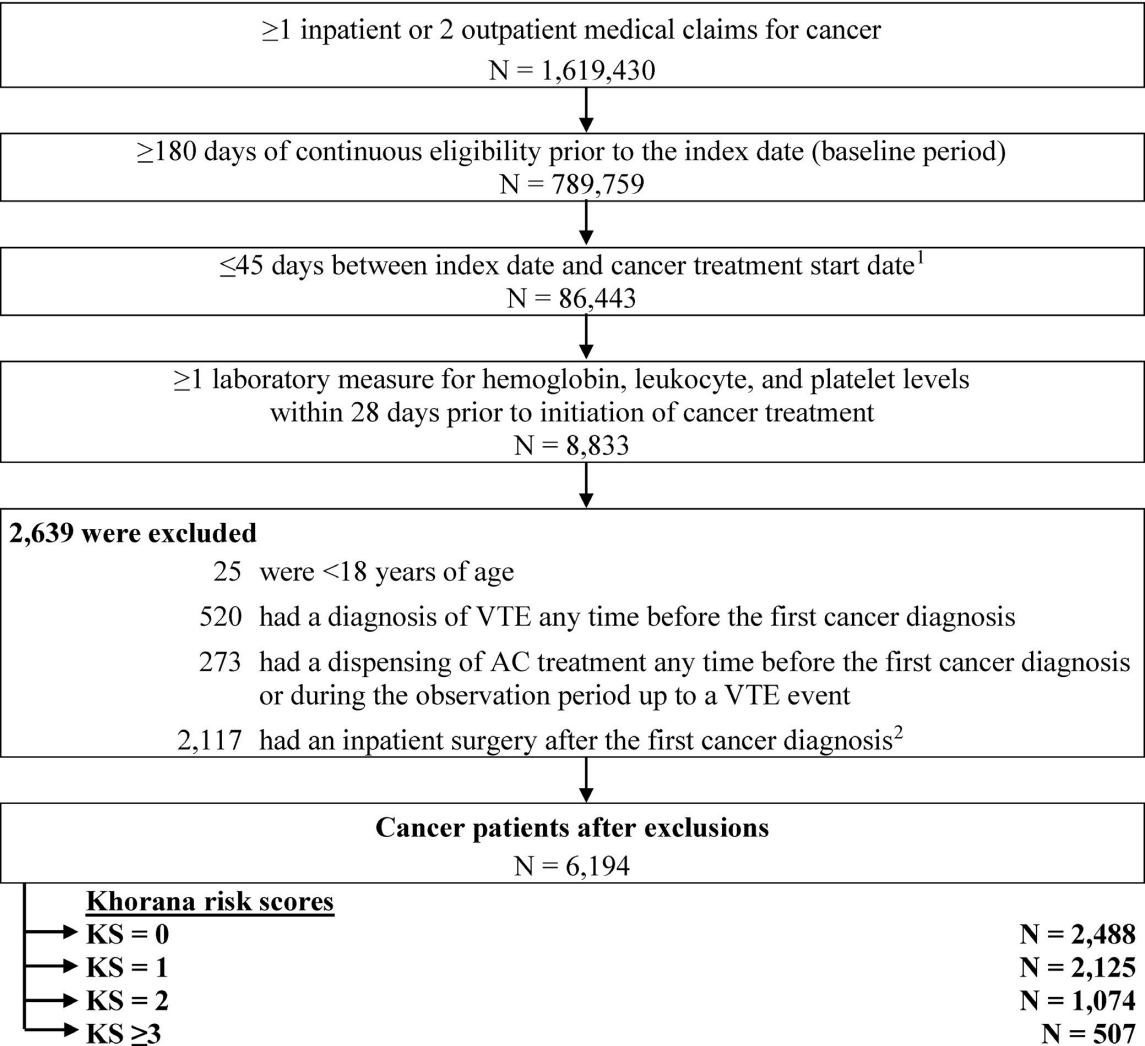
Patient Disposition. Abbreviations: AC, anticoagulant; AF, atrial fibrillation; VTE, venous thromboembolism. Notes: 1. Cancer treatment included non‐radiation and radiation therapies. 2. Defined as a procedure code for major surgery, abdominopelvic surgery, and neurosurgery or orthopedic surgery during an inpatient stay

Patients were included in the analysis if they met the following criteria: (1) age at index ≥ 18 years; (2) ≥ 2 outpatient visits or ≥ 1 hospitalization with a diagnosis of cancer (ICD‐9‐CM: 140‒209; ICD‐10‐CM: C00‒C7A); (3) having initiated chemotherapy (based on the list from the National Cancer Institute^39^) or radiation therapy ≤ 45 days post‐index date; (4) ≥ 6 months of continuous eligibility during the baseline period; and (5) had ≥ 1 laboratory test result for hemoglobin, platelet, and leukocyte counts during the 28‐day risk stratification period **(**Figure 1).

The following exclusion criteria were applied: (1) had a diagnosis of VTE during the 6‐month baseline period; (2) used anticoagulants any time prior to the index date or during the follow‐up period; or (3) had an inpatient surgery after the first cancer diagnosis, defined as a procedure code for abdominopelvic surgery, major surgery, and neurosurgery or orthopedic surgery during an inpatient stay (**Figure** **1**).

Patients who met all inclusion and exclusion criteria were further stratified into four mutually exclusive cohorts based on their KS. More specifically, the KS was calculated based on the cancer's primary site at the time of initial diagnosis, BMI, and lab values for hemoglobin, platelet, and leukocyte count during the risk stratification period (28 days prior to the treatment start date). ICD‐9‐CM and ICD‐10‐CM diagnostic codes were used as a proxy for body mass index (BMI) ≥ 35 in the KS calculation.

The present study population comprised all patients with cancer. A sensitivity analysis was conducted among the subgroup of patients with the same tumor types (ie breast, lung, ovarian, sarcoma, colon, and lymphomas) that were used to develop the KS.^24^

### 2.4 Study outcome

Study outcomes included VTE events and survival. VTEs were evaluated during the follow‐up period for each KS cohort and were defined as (1) a diagnosis of VTE in the primary position during a hospitalization, or (2) a hospitalization with a medical claim for a secondary diagnosis of VTE followed by an anticoagulant dispensing or administration ≤ 30 days post‐discharge of the first hospitalization, or another hospitalization or an outpatient visit with a primary or secondary VTE diagnosis, or (3) a primary or secondary outpatient VTE diagnosis followed by an anticoagulant dispensing or administration within 30 days after the outpatient visit. Survival was assessed among patients with vs without VTE during the follow‐up period for each KS cohort.

### 2.5 Statistical analysis

Baseline characteristics, overall and stratified by KS risk cohorts, were reported using descriptive statistics. More specifically, frequencies and proportions were used to summarize categorical variables; means, standard deviations, and medians were used to summarize continuous variables.

Time to first VTE event was evaluated using Kaplan‐Meier (KM) analyses. Censoring was applied to patients without a VTE at the end of the observation period. KM rates of VTE were reported at three, six, nine, and 12 months and were compared between KS cohorts using hazard ratios from Cox proportional hazards models adjusting for the following covariates evaluated during the baseline period or on the index date: sex, age, type of insurance plan, region, year/month of index date, Elixhauser comorbidities with a proportion ≥ 5%,^40^ Charlson comorbidity index (CCI) score, healthcare resource utilization, and healthcare costs. Furthermore, the mean and median time to VTE was also reported.

Survival at 12 months of follow‐up was also evaluated using KM analyses. Adjusted hazard ratios from Cox proportional hazard models (controlling for the same covariates used for the analysis of VTE rates) were used to compare survival between patients with and without VTE events.

## 3 RESULTS

A total of 6194 cancer patients met all inclusion criteria. Of these, 2488 (40.2%) were classified in the KS = 0 cohort, 2125 (34.3%) in the KS = 1 cohort, 1074 (17.3%) in the KS = 2 cohort, and 507 (8.2%) in the KS ≥ 3 cohort (Table 1 and Figure 1).

**Table 1:** Demographic and clinical characteristics

Baseline Characteristics	All patients N = 6194	Khorana Score^a^			
		KS = 0	KS = 1	KS = 2	KS ≥ 3
		N = 2488	N = 2125	N = 1074	N = 507
Eligibility post‐index, months, mean ± SD [median]	17.1 ± 15.1 [12.1]	20.5 ± 16.0 [15.8]	16.6 ± 14.9 [11.6]	13.4 ± 12.9 [8.8]	10.4 ± 10.8 [6.4]
Observation period^b^, months, mean ± SD [median]	8.8 ± 3.9 [12.0]	9.6 ± 3.6 [12.0]	8.7 ± 3.9 [11.6]	8.0 ± 4.0 [8.8]	6.9 ± 4.2 [6.4]
Demographics					
Age^c^, mean ± SD [median]	68.0 ± 12.2 [70]	67.6 ± 12.2 [69]	68.2 ± 12.5 [70]	68.3 ± 12.2 [70]	68.4 ± 11.0 [70]
Gender^c^, female, n (%)	3021 (48.8)	1185 (47.6)	1032 (48.6)	553 (51.5)	251 (49.5)
Year of index date, n (%)					
2012	408 (6.6)	185 (7.4)	143 (6.7)	55 (5.1)	25 (4.9)
2013	1212 (19.6)	512 (20.6)	409 (19.2)	197 (18.3)	94 (18.5)
2014	1127 (18.2)	480 (19.3)	368 (17.3)	176 (16.4)	103 (20.3)
2015	1228 (19.8)	491 (19.7)	407 (19.2)	226 (21.0)	104 (20.5)
2016	1273 (20.6)	479 (19.3)	443 (20.8)	246 (22.9)	105 (20.7)
2017	946 (15.3)	341 (13.7)	355 (16.7)	174 (16.2)	76 (15.0)
Region^c^, n (%)					
South	2435 (39.3)	1020 (41.0)	823 (38.7)	405 (37.7)	187 (36.9)
West	2244 (36.2)	907 (36.5)	784 (36.9)	390 (36.3)	163 (32.1)
Midwest	762 (12.3)	259 (10.4)	264 (12.4)	152 (14.2)	87 (17.2)
Northeast	712 (11.5)	285 (11.5)	238 (11.2)	123 (11.5)	66 (13.0)
Unknown	41 (0.7)	17 (0.7)	16 (0.8)	4 (0.4)	4 (0.8)
Insurance plan type^c^, n (%)					
Medicare	4144 (66.9)	1578 (63.4)	1476 (69.5)	733 (68.2)	357 (70.4)
Point‐of‐service	1402 (22.6)	640 (25.7)	419 (19.7)	233 (21.7)	110 (21.7)
Health maintenance organization	346 (5.6)	147 (5.9)	115 (5.4)	59 (5.5)	25 (4.9)
Exclusive provider organization	255 (4.1)	104 (4.2)	99 (4.7)	38 (3.5)	14 (2.8)
Preferred provider organization	24 (0.4)	13 (0.5)	8 (0.4)	3 (0.3)	0 (0.0)
Indemnity	23 (0.4)	6 (0.2)	8 (0.4)	8 (0.7)	1 (0.2)
**Charlson comorbidity index** ^d^ **, mean ± SD [median]**	1.2 ± 1.5 [1]	1.1 ± 1.4 [1]	1.3 ± 1.5 [1]	1.4 ± 1.5 [1]	1.4 ± 1.6 [1]
Cancer type^c^, n (%)					
*Solid cancers*	4737 (76.5)	2008 (80.7)	1480 (69.6)	815 (75.9)	434 (85.6)
Lung	1042 (16.8)	0 (0.0)	505 (23.8)	334 (31.1)	203 (40.0)
Prostate	630 (10.2)	509 (20.5)	102 (4.8)	19 (1.8)	0 (0.0)
Breast	885 (14.3)	643 (25.8)	193 (9.1)	43 (4.0)	6 (1.2)
Colorectal	179 (2.9)	70 (2.8)	58 (2.7)	38 (3.5)	13 (2.6)
Other solid cancer	2025 (32.7)	787 (31.6)	634 (29.8)	388 (36.1)	216 (42.6)
*Hematologic cancers*	1439 (23.2)	266 (10.7)	723 (34.0)	335 (31.2)	115 (22.7)
Type of index cancer treatment, n (%)					
Chemotherapy	3861 (62.3)	1383 (55.6)	1405 (66.1)	739 (68.8)	334 (65.9)
Radiation therapy	2370 (38.3)	1120 (45.0)	732 (34.4)	340 (31.7)	178 (35.1)
All‐cause HRU^d^, mean ± SD [median]					
*All‐cause*					
Hospitalizations	0.10 ± 0.36 [0]	0.06 ± 0.30 [0]	0.11 ± 0.36 [0]	0.14 ± 0.46 [0]	0.12 ± 0.36 [0]
ER visits	0.43 ± 1.24 [0]	0.36 ± 1.20 [0]	0.45 ± 1.29 [0]	0.50 ± 1.21 [0]	0.48 ± 1.23 [0]
Outpatient visits	7.34 ± 8.17 [6]	6.88 ± 6.91 [5]	7.89 ± 9.08 [6]	7.65 ± 8.80 [5]	6.67 ± 8.37 [5]
Other visits	2.96 ± 6.10 [0]	2.57 ± 5.74 [0]	3.17 ± 6.12 [0]	3.25 ± 6.48 [0]	3.34 ± 6.75 [0]
All‐cause healthcare cost^d^, US$ 2018, mean ± SD					
*All‐cause*					
Total healthcare costs	8689 ± 17 848	7864 ± 18 334	9352 ± 17 999	9542 ± 16 564	8147 ± 17 254
Hospitalization costs	1554 ± 8943	1061 ± 7179	1683 ± 8032	2226 ± 11 464	2002 ± 13 181
ER visit costs	1174 ± 5752	1004 ± 6946	1207 ± 4589	1430 ± 4905	1330 ± 5304
Outpatient visit costs	3850 ± 11 161	3643 ± 11 304	4378 ± 13 240	3719 ± 7593	2929 ± 6010
Other medical costs	914 ± 3148	825 ± 2957	959 ± 3038	1040 ± 3,827	896 ± 2907
Pharmacy costs	1197 ± 5019	1331 ± 6633	1124 ± 3922	1127 ± 3218	990 ± 2313

Abbreviations: ER, emergency room; HRU, healthcare resource utilization; SD, standard deviation.

^a^ Patients are stratified by Khorana score, which was calculated using the site of first cancer diagnosis, body mass index, and lab values of hemoglobin, leukocyte, and platelet tests within 28 days back from the treatment start date.

^b^ The observation period was censored at 12 month for patients with ≥ 1 year of eligibility post‐index.

^c^ Evaluated at the index date.

^d^ Evaluated during the 6‐month baseline period.

### 3.1 Baseline characteristics

Overall, patients were on average 68.0 years old, had good geographical representation across the four US census regions, and 48.8% were female (Table 1). Most patients (76.5%) were diagnosed with solid tumors. Lung (16.8%), breast (14.3%), and prostate cancer (10.2%) were the most common malignancies (Table 1). A majority of patients (62.3%) received chemotherapy as index cancer treatment (Table 1).

Age, sex, and region of residence did not vary substantially across the different KS cohorts (Table 1). The mean CCI was 1.2 in the overall cohort and did not differ across the KS cohorts (Table 1). The mean durations of the observation period ranged from 9.6 months in the KS = 0 cohort to 6.9 months in the KS ≥ 3 cohort (Table 1).

### 3.2 Rate of VTE events associated with different Khorana scores

**Figure 2:**
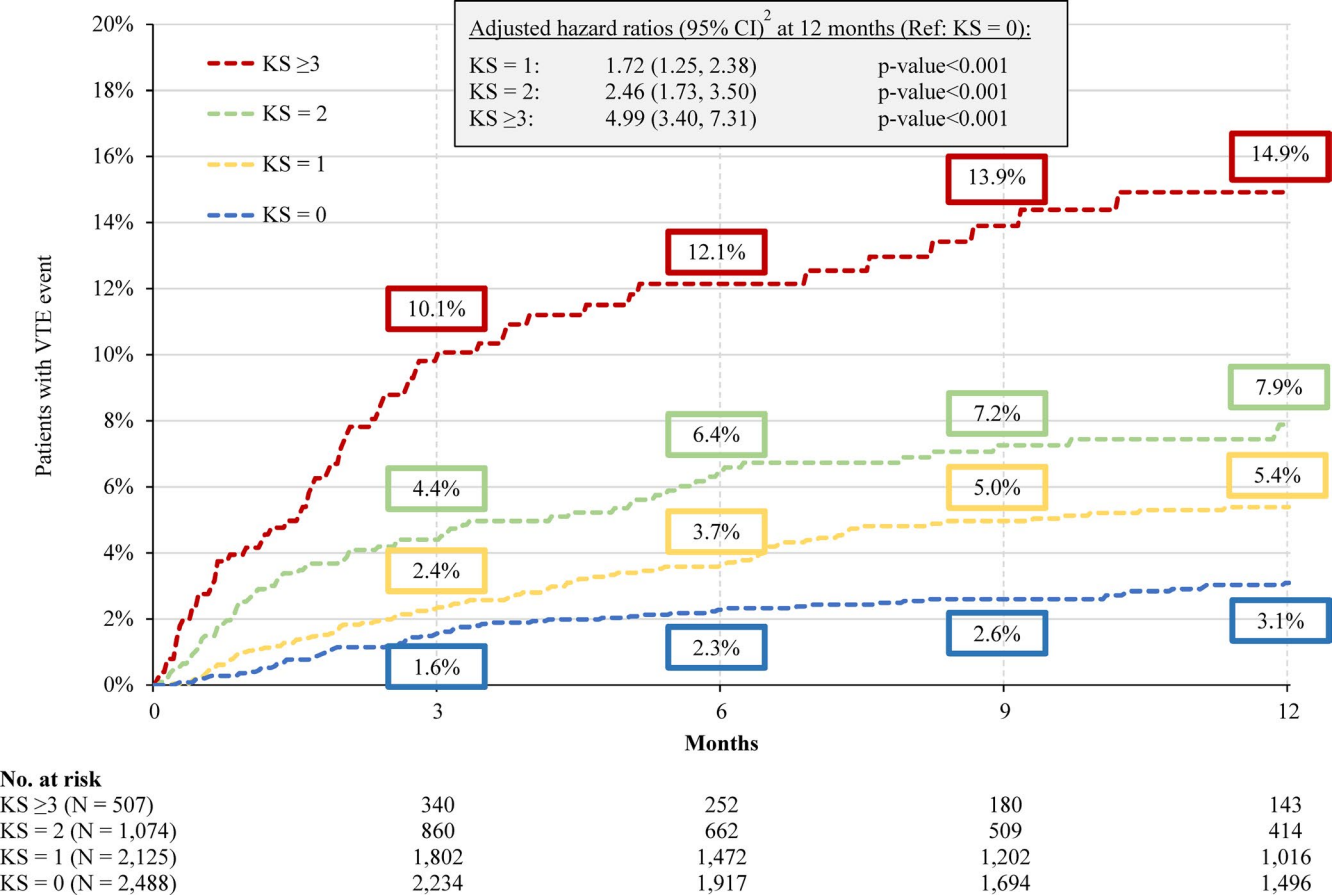
Kaplan‐Meier Rates of VTE^1^ up to 12 Months of Follow‐up. Abbreviations: CI, confidence interval; KS, Khorana score; VTE, venous thromboembolism. Notes: 1. A VTE was defined as (1) a primary VTE diagnosis during a hospitalization or (2) a secondary VTE diagnosis during a hospitalization followed by another hospitalization or outpatient diagnosis or an anticoagulant dispensing within 30 days after the first hospitalization discharge date or (3) a primary or secondary outpatient VTE diagnosis followed by an AC dispensing or administration within 30 days after the outpatient visit. 2. Hazard ratio is calculated using a Cox proportional hazard model adjusting for sex, age, index year, region, insurance type, Charlson comorbidity index score, baseline healthcare resource use and costs, and comorbidities with a proportion ≥ 5%

Over the first three months of follow‐up, the KM rate of VTE sharply increased to 10.1% for patients in the KS ≥ 3 cohort compared with 4.4%, 2.4%, and 1.6% for patients with KS = 2, 1, and 0, respectively (Figure 2). The KM rate of VTE in the KS ≥ 3 cohort reached 12.1% at six months, 13.9% at nine months, and 14.9% at 12 months; 32.4% of all VTEs occurred after the first three months post‐index date. For patients in the KS = 2 cohort, the KM rate of VTE reached 6.4% at six months, 7.2% at nine months, and 7.9% at 12 months of follow‐up; 44.1% of all VTEs occurred after the initial three months of follow‐up. Similar trends were found in the KS = 1 and KS = 0 cohorts, but absolute increases in the KM rate of VTE were lower (ie, 12‐month VTE rates of 5.4% and 3.1%, respectively). Moreover, the associated mean (median) time to VTE event was 3.8 (2.7), 3.6 (3.0), 2.7 (1.4), and 2.3 (1.7) months for the KS = 0, 1, 2, and ≥ 3 cohorts, respectively.

When comparing the risk of VTE event across the four KS cohorts using the KS = 0 cohort as reference, the risk of VTE was nearly five times higher in the KS ≥ 3 cohort (HR [95% CI] =4.99 [3.40‐7.31], *P* < .001; Figure 2). For patients in the KS = 2 and KS = 1 cohorts, this risk was 2.46 (95% CI = 1.73‐3.50, *P* < .001) and 1.72 (95% CI = 1.25‐2.38, *P* < .001) times higher, respectively, compared to KS = 0 (Figure 2).

**Figure 3:**
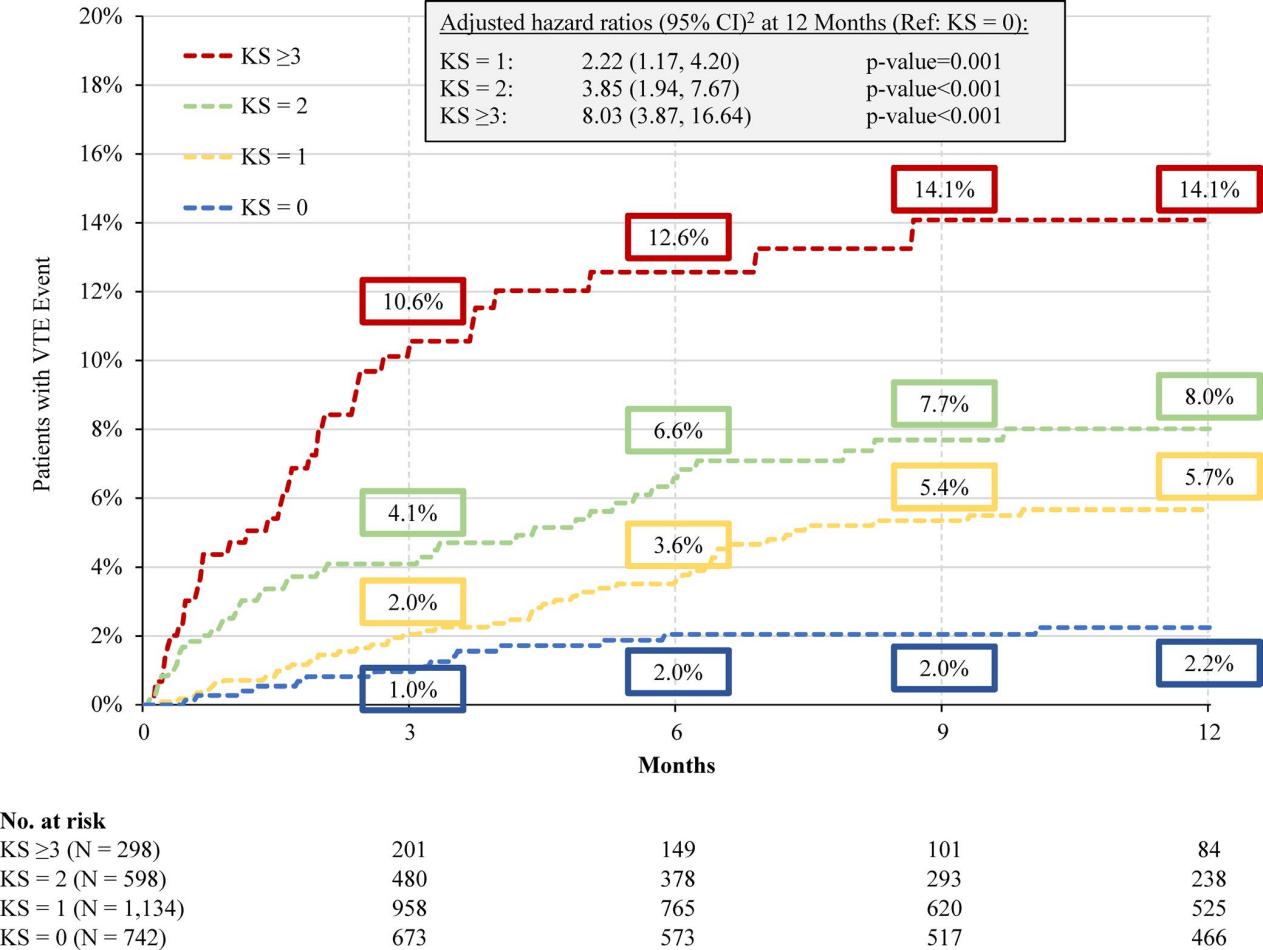
Kaplan‐Meier Rates of VTE^1^ up to 12 Months of Follow‐up – Original KS Cancers Subgroup Abbreviations: CI, confidence interval; KS = Khorana score; VTE, venous thromboembolism Notes: 1. A VTE was defined as (1) a primary VTE diagnosis during a hospitalization or (2) a secondary VTE diagnosis during a hospitalization followed by another hospitalization or outpatient diagnosis or an anticoagulant dispensing within 30 days after the first hospitalization discharge date or (3) a primary or secondary outpatient VTE diagnosis followed by an AC dispensing or administration within 30 days after the outpatient visit. 2. Hazard ratio is calculated using a Cox proportional hazard model adjusting for sex, age, index year, region, insurance type, Charlson comorbidity index score, baseline healthcare resource use and costs, and comorbidities with a proportion ≥ 5%

Results were similar among the subgroup of 2772 patients with the same tumor types as in the original KS. The KM rates of VTE at 12 months of follow‐up were 2.2%, 5.7%, 8.0%, and 14.1% in the KS = 0, 1, 2, and ≥ 3 cohorts, respectively (Figure 3). Relative to the KS = 0 cohort, the risk of VTE was eight times higher in the KS ≥ 3 cohort (HR [95% CI] = 8.03 [3.87‐16.64], *P* < .001), almost four times higher in the KS = 2 cohort (HR [95% CI] =3.85 [1.94‐7.67], *P* < .001), and two times higher in the KS = 1 cohort (HR [95% CI] =2.22 [1.17‐4.20], *P* < .001; Figure 3).

### 3.3 Survival rate associated with different Khorana scores

**Figure 4:**
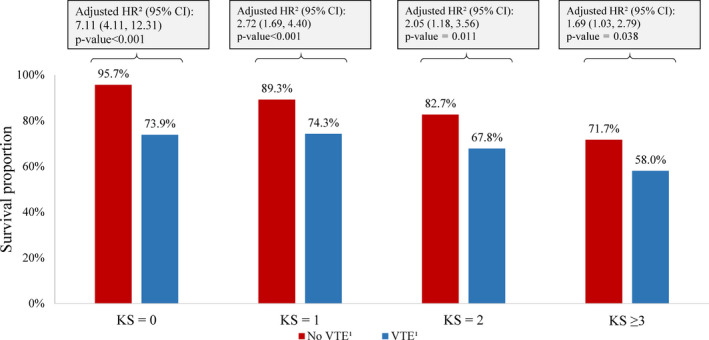
Kaplan‐Meier Survival Rates up to 12 Months of Follow‐up. Abbreviations: CI, confidence interval; HR, hazard ratio; KS, Khorana score; VTE, venous thromboembolism. Notes: 1. A VTE event is defined as (1) a primary inpatient VTE diagnosis or (2) a secondary inpatient VTE diagnosis followed by another inpatient or outpatient diagnosis or an AC dispensing within 30 days after discharge date or (3) a primary or secondary outpatient VTE diagnosis followed by an AC dispensing or administration within 30 days. 2. Hazard ratios were calculated using Cox proportional hazards models adjusting for sex, age, index year, region, insurance type, Charlson comorbidity index score, baseline healthcare resource use and costs, and Elixhauser comorbidities with a proportion ≥ 5%

Overall, survival rates decreased with increasing risk of VTE. Relative to patients with VTE, survival rates were significantly higher among patients without VTE in the KS = 0 (HR [95% CI] = 7.11 [4.11‐12.31], *P* < .001), KS = 1 (HR [95% CI] =2.72 [1.69‐4.40], *P* < .001), KS = 2 (HR [95% CI] =2.05 [1.18‐3.56], *P* = .011), and KS ≥ 3 (HR [95% CI] =1.69 [1.03‐2.79], *P* = .038) cohorts at 12 months of follow‐up (Figure 4). Patients in the KS = 0 and KS = 1 cohorts who did not experience a VTE event had higher survival rates (95.7% and 89.3%, respectively) than those in the KS = 2 and KS ≥ 3 cohorts (82.7% and 71.7%, respectively; Figure 4). Among patients who experienced a VTE event, survival rates were similar between patients classified in the KS = 0 and KS = 1 cohorts (73.9% and 74.3%, respectively), but lower for patients in KS = 2 and KS ≥ 3 cohorts (67.8% and 58.0%, respectively; Figure 4).

**Figure 5:**
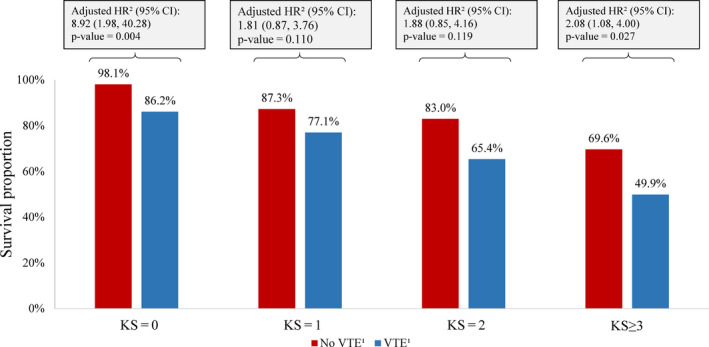
Kaplan‐Meier Survival Rates up to 12 Months of Follow‐up – Original KS Cancers Subgroup. Abbreviations: CI, confidence interval; HR, hazard ratio; KS, Khorana score; VTE, venous thromboembolism. Notes: 1. A VTE event is defined as (1) a primary inpatient VTE diagnosis or (2) a secondary inpatient VTE diagnosis followed by another inpatient or outpatient diagnosis or an AC dispensing within 30 days after discharge date or (3) a primary or secondary outpatient VTE diagnosis followed by an AC dispensing or administration within 30 days. 2. Hazard ratios were calculated using Cox proportional hazards models adjusting for sex, age, index year, region, insurance type, Charlson comorbidity index score, baseline healthcare resource use and costs, and Elixhauser comorbidities with a proportion ≥ 5%

When limiting to patients with specific tumor types (ie breast, lung, ovarian, sarcoma, colon, lymphomas), patients without vs with VTE also had greater survival among patients with KS = 0 (HR [95% CI] =8.92 [1.98‐40.28], *P* = .004), KS = 1 (HR [95% CI] =1.81 [0.87‐3.76], *P* = .110), KS = 2 (HR [95% CI] =1.88 [0.85‐4.16], *P* = .119), and KS ≥ 3 (HR [95% CI] =2.08 [1.08‐4.00], *P* = .027; Figure 5).

## 4 DISCUSSION

In this retrospective cohort study using real‐world data, the rates of VTE were evaluated in cancer patients stratified by their risk of VTE using the KS in a large US claims database. Over 25% of cancer patients had a KS of ≥ 2, and these patients were approximately three times more likely to develop a VTE compared to patients with KS = 0. Patients who developed VTE had a significantly higher risk of death than those without a VTE, regardless of the KS cohort. Among patients who subsequently had a VTE, only 67.8% of those with KS = 2 and 58.0% of those with KS ≥ 3 were still alive at 12 months of follow‐up. Study findings were similar when evaluating patients with the same cancer types as the original KS (ie breast, lung, ovarian, sarcoma, colon, and lymphomas).^24^

Until recently, most guidelines did not recommend primary VTE prophylaxis in outpatients with cancer,^27^, ^41^ even though VTE incidence rates are very high in this patient population.^42^ This was in part because the numbers needed to treat (NNTs) reported in the PROTECHT^35^ and SAVE‐ONCO^34^ trials were high, so the benefits of thromboprophylaxis were perceived as low.^8^, ^34^, ^35^, ^43^ Subsequent post‐hoc analyses demonstrated that these NNTs can be reduced by focusing on a subset of patients at high risk of VTE using the KS. Indeed, subgroup analyses of high‐risk patients with KS ≥ 3 enrolled in PROTECHT and SAVE‐ONCO found NNTs of 15 and 25, respectively.^23^, ^31^ Furthermore, a trial in which high‐risk cancer patients with KS ≥ 3 were treated with LMWH reported an NNT of 11.^32^ More recently, the CASSINI (rivaroxaban vs placebo)^36^ trial reported an NNT of 26, and the AVERT (apixaban vs placebo)^37^ trial reported an NNT of 16 during the on‐treatment period in patients with cancer and a KS ≥ 2. Based on this body of evidence, recent updates of the ASCO and ISTH guidelines stipulate that primary VTE prophylaxis can be considered in high‐risk ambulatory patients with cancer without contraindications. The results of the present study add to the data generated by the aforementioned studies by documenting the prognosis of patients in higher KS categories in a real‐world setting. The high VTE rates and mortality observed in the current study emphasize the unmet clinical needs of these patients.

Current guidelines from the National Comprehensive Cancer Network (NCCN) and the ASCO recommend primary VTE prophylaxis in most inpatients with cancer.^27^, ^28^ However, the available evidence supporting this recommendation is derived from studies of general medical and surgical patient populations. One meta‐analysis of three trials reported efficacy results for subgroups of patients with cancer, but the risk of VTE was not significantly reduced (relative risk [95% CI] =0.91 [0.21‐4.0]).^44^ Furthermore, results from Zwicker *et al* suggest that risk factors are not taken into account when prescribing VTE prophylaxis for hospitalized patients with cancer, with 79% of high‐risk patients and 63% of low‐risk patients receiving primary VTE prophylaxis.^45^ The potential harms of anticoagulation, such as the risk of major bleeding, may outweigh their benefits in a low‐risk population, hence the importance of having validated tools to target patients who may benefit the most from anticoagulation. The data from the current study emphasizes that the use of a validated tool such as the KS in both inpatient and outpatient settings is one means to refine the current approach described by Zwicker *et al*

In this study, the risk of VTE most sharply increased during the first three months after cancer diagnosis in all KS cohorts, confirming previous observations that the risk of VTE is highest during the first few months after cancer diagnosis.^1^, ^46^ However, the risk did not plateau beyond those three months similar to other real‐world cancer‐associated VTE studies,^47^ with about 40% of all VTEs occurring from months three to 12 in the KS = 2 and KS ≥ 3 cohorts. This suggests that cancer patients with KS ≥ 3 remain at risk for VTE beyond the initial three months and that prolonged thromboprophylaxis may be beneficial in these patients, although this intervention has not been tested in the current study. Notably, the three‐month period roughly corresponds to the follow‐up time in the SAVE‐ONCO and PROTECHT trials,^34^, ^35^ suggesting the absolute VTE rates in these trials might have been higher over longer follow‐up. Further research is warranted to assess the potential benefits of prolonged thromboprophylaxis beyond three months.

### 4.1 Limitations

The current study is subject to some limitations. First, included patients were treated with either chemotherapy or RT, and results may not apply to patients receiving other cancer treatments. Second, this claims‐based study may also be subject to residual confounding due to unmeasured confounders, such as cancer stage. Third, BMI was evaluated using insurance claims with BMI ICD codes, which may lead to underestimation of the KS. Fourth, in spite of the large size of the present study population and its nationwide geographical representation, findings may not be generalizable to patients with health insurance plans that substantially differ from those of the population analyzed in this study. Finally, healthcare claims may contain coding omissions and inaccuracies that may influence the absolute VTE rates reported. Our conservative approach, requiring at least two diagnostic codes or a diagnostic code and anticoagulant code makes underreporting of VTEs possible, but over‐reporting unlikely.

## 5 CONCLUSIONS

In this large real‐world retrospective analysis, over 25% of patients diagnosed with cancer had a KS of ≥ 2 (high risk of VTE), and these patients were three times more likely to develop VTE than patients with KS = 0 (low risk of VTE) at the time of their cancer diagnosis. Survival was particularly low among patients in higher KS categories, which might be explained by different types of cancer and advanced stages of cancer. Interestingly, we observed that among patients at low risk for VTE (KS = 0), those developing VTE are approximately seven times more likely to die prematurely relative to those who do not develop VTE. Moreover, patients at higher risk of VTE (KS ≥ 2) who developed VTE were approximately twice as likely to die relative to patients who did not develop VTE over 12 months of follow‐up. These results build on those of previous clinical studies by documenting the prognosis of patients falling into different risk categories in a real‐world setting. In light of the recent recommendation to offer primary VTE prophylaxis to high‐risk ambulatory patients with cancer,^28^, ^29^ future studies are warranted to determine the impact of primary VTE prophylaxis on patient survival in real‐world settings.

## PREVIOUS PRESENTATION

Parts of the finding in this manuscript was presented at the 60th American Society of Hematology (ASH) annual meeting held December 1‐4, 2018 in San Diego, CA, and 61st ASH held December 7‐10, 2019 in Orlando, Florida.

## ETHICAL APPROVAL STATEMENT

The data for study participants were de‐identified and complied with the Health Insurance Portability and Accountability Act (HIPAA); therefore, no reviews by an institutional review board were required.

## CONFLICT OF INTEREST

GG, FL, SDM, and PL are employees of Groupe d'analyse, Ltée, a consulting company that provided paid consulting services to Janssen Scientific Affairs, LLC. for the conduct of the present study. DM is an employee of Janssen Scientific Affairs and shareholders of Johnson & Johnson. AAK, NMK, KMC, GHL, and MBS have received research funds from Janssen Scientific Affairs.

## AUTHOR CONTRIBUTION

All authors were responsible for the study design and the interpretation of the study results. FL, GG, and SDM were responsible for the data analysis. All authors reviewed the manuscript for intellectual content and approved the final draft.

## Data Availability

This study used claims data to analyze the risk of venous thromboembolism and mortality in patients with cancer. The claims database (Optum's Clinformatics^®^ Data Mart Databases [Optum databases]) is provided by Optum, which was as a third‐party vendor. Janssen Scientific Affairs purchased access to the Optum databases (on a contract per‐project use). Other interested parties may also access this data set by contacting Optum (https://www.optum.com/contact.html).
